# Malaria severity: Possible influence of the E670G PCSK9 polymorphism: A preliminary case-control study in Malian children

**DOI:** 10.1371/journal.pone.0192850

**Published:** 2018-02-15

**Authors:** Charles Arama, Issa Diarra, Bourèma Kouriba, Francine Sirois, Olesya Fedoryak, Mahamadou A. Thera, Drissa Coulibaly, Kirsten E. Lyke, Christopher V. Plowe, Michel Chrétien, Ogobara K. Doumbo, Majambu Mbikay

**Affiliations:** 1 Malaria Research and Training Center, International Centers for Excellence in Research, Université des Sciences, des Techniques et des Technologies de Bamako, Bamako, Mali; 2 Laboratoire de protéolyse fonctionnelle, Institut de recherches cliniques de Montréal, Montréal, Québec, Canada; 3 Center for Vaccine Development and Division of Malaria Research, Institute for Global Health, University of Maryland School of Medicine, Baltimore, Maryland, United States of America; 4 Chronic Disease Program, Ottawa Hospital Research Hospital, Ottawa, Ontario, Canada; 5 Department of Biochemistry, Microbiology, and Immunology, Faculty of Medicine, University of Ottawa, Ottawa, Ontario, Canada; Hokkaido Daigaku, JAPAN

## Abstract

**Aim:**

Proprotein Convertase Subtilisin/Kexin Type 9 (PCSK9) is a hepatic secretory protein which promotes the degradation of low-density lipoprotein receptors leading to reduced hepatic uptake of plasma cholesterol. Non-synonymous single-nucleotide polymorphisms in its gene have been linked to hypo- or hyper- cholesterolemia, depending on whether they decrease or increase PCSK9 activity, respectively. Since the proliferation and the infectivity of *Plasmodium spp*. partially depend on cholesterol from the host, we hypothesize that these *PCSK9* genetic polymorphisms could influence the course of malaria infection in individuals who carry them. Here we examined the frequency distribution of one dominant (C679X) and two recessive (A443T, I474V) hypocholesterolemic polymorphisms as well as that of one recessive hypercholesterolemic polymorphism (E670G) among healthy and malaria-infected Malian children.

**Methods:**

Dried blood spots were collected in Bandiagara, Mali, from 752 age, residence and ethnicity-matched children: 253 healthy controls, 246 uncomplicated malaria patients and 253 severe malaria patients. Their genomic DNA was extracted and genotyped for the above *PCSK9* polymorphisms using Taqman assays. Associations of genotype distributions and allele frequencies with malaria were evaluated.

**Results:**

The minor allele frequency of the A443T, I474V, E670G, and C679X polymorphisms in the study population sample was 0.12, 0.20, 0.26, and 0.02, respectively. For each polymorphism, the genotype distribution among the three health conditions was statistically insignificant, but for the hypercholesterolemic E670G polymorphism, a trend towards association of the minor allele with malaria severity was observed (*P* = 0.035). The association proved to be stronger when allele frequencies between healthy controls and severe malaria cases were compared (Odd Ratio: 1.34; 95% Confidence Intervals: 1.04–1.83); *P* = 0.031).

**Conclusions:**

Carriers of the minor allele of the E670G PCSK9 polymorphism might be more susceptible to severe malaria. Further investigation of the cholesterol regulating function of PCSK9 in the pathophysiology of malaria is needed.

## Introduction

Proprotein Convertase Subtilisin/Kexin Type 9 (PCSK9) is a secretory glycoprotein discovered in 2003 and initially termed Neural Apoptosis-Regulated Convertase 1 (NARC-1) [1]. It belongs to the family of proprotein convertases, the serine endoproteinases involved in the proteolytic activation of a variety of secretory precursor proteins [2, 3]. Its gene is located on human chromosome 1. It was the segregation of missense mutations at its locus with autosomal dominant hypercholesterolemia (ADH) that led to the identification of its function in cholesterol metabolism [4].

PCSK9 is primarily expressed in the liver. It is biosynthesized as a 692-amino acid preproPCSK9 within hepatocytes and cleaves itself after the prodomain, forming an enzymatically inactive heteroduplex made of the propeptide and the mature PCSK9. After release into the bloodstream, it acts as an escort protein for low-density lipoprotein receptor (LDLR) to which it binds at the surface of hepatocytes; the binding pair is internalized and directed towards lysosome-like compartments where it is degraded [5]. LDLR mediates hepatic uptake of plasma LDL and thus contributes to the clearance of plasma cholesterol [6]. PCSK9 genetic mutations that strongly enhance its LDLR-degrading activity of the protein have been implicated in autosomal dominant hypercholesterolemia [4, 7]. Furthermore, epidemiological studies have revealed strong associations between several nonsynonymous PCSK9 single-nucleotide polymorphisms (SNPs) with hyper- or hypo-cholesterolemia in humans [8–14]. Gain-of-function (GOF) SNPs accentuate PCSK9 activity, leading to hypercholesterolemia whereas loss-of-function (LOF) SNPs attenuate the activity, leading to hypocholesterolemia. Hypercholesterolemia is a risk factor for atherosclerosis and other cardiovascular diseases [15]. PCSK9 inhibitors have proven to be effective drugs against this condition [16, 17].

Two of the most effective LOF *PCSK9* SNPs are c.426C> G and c.2037C> A, which cause the nonsense p.Y142X and p.C679X mutations, respectively. The two mutations are found at a rate of 1 in 40 African Americans and are 100-fold less frequent in European Americans [8]. In a large retrospective study, heterozygosity for these mutations was associated with lifelong hypocholesterolemia and significant protection against coronary heart disease (CHD) [18]. In West African ethnic groups the minor allele frequency (MAF) of the C679X mutation averages 3.3% overall but ranges from 0 to 7% [19].

Until recently, CHD was relatively uncommon in sub-Saharan Africa, primarily because of lower prevalence of lifestyle risk factors [20]. Although it is possible that these cardioprotective PCSK9 mutations, may have also contributed to the past low occurrence of CHD, it is probable that these mutations may have been maintained at high frequency in these populations because they conferred some protection against major causes of mortality before reproductive age. We hypothesize that these causes might have been infectious diseases, malaria in particular [21]. This hypothesis was based on the mounting evidence that host cholesterol significantly contributes to invasion and proliferation of infectious agents [22, 23]. In the case of malaria, studies have shown that hypocholesterolemia conferred protection against malaria by reducing the infectivity of merozoites [24, 25]. Furthermore, the *Plasmodium* parasite scavenges endogenously produced or LDLR-captured cholesterol for its proficient replication in hepatocytes [26]. Merozoites also need cholesterol for development within the erythrocytes, suggesting that any alteration in cholesterol mobilization and metabolism may influence parasite development, and explaining the fact that reduction of plasma lipoprotein-cholesterol has been observed in individuals with acute malaria [27]. From a therapeutic angle, it has been reported that Atorvastatin, a member of the statin-family of anti-cholesterol drugs, can potentiate the efficacy of standard anti-malarial drug such mefloquine and artemisinin in murine model of cerebral malaria [28, 29].

We wanted to assess whether by affecting cholesterol metabolism, PCSK9 could influence the pathophysiology of malaria. As a first step, we have determined the frequency of four common *PCSK9* SNPs with a documented cholesterolemia phenotype in sub-Saharan African children of Mali enrolled in a case-control study evaluating risk and protective factors for severe malaria.

## Materials and methods

### Ethics

The study protocol was reviewed and approved by the Review Board of the University of Mali Faculty of Medicine, Pharmacy and Dentistry as well as the Research Ethics Committee of the Clinical Research Institute of Montreal. The original case control study protocol and the use of archived samples for malaria studies was approved by the University of Maryland Baltimore Institutional Review Board. Written informed consent was obtained from parents or guardians of all study participants.

### Subjects

The subjects were Malian children (n = 752), aged 3 months to 14 years, enrolled in a case-control study evaluating risk and protective factors for severe malaria [30]. Index cases of severe malaria (SM, n = 253) from Bandiagara and surrounding areas were enrolled from July 2000 to December 2001. In all three groups, the mean age was about 40 months, 85% of children were aged less than 5½ years, and nearly 80% of them belonged to the Dogon ethnic group (Supporting information: S1 Table). Cases were classified as severe malaria based on the World Health Organization criteria which include one or more of the following symptoms: impaired consciousness, prostration, multiple convulsions, acidosis, hypoglycemia, renal impairment, severe anemia, jaundice, pulmonary edema, shock, or hyperparasitemia [31]. Each index case was age-, residence-, and ethnicity-matched to a case of uncomplicated malaria (UM) and a healthy control (HC). UM was defined as *P*. *falciparum* parasitemia and an axillary temperature ≥ 37.5 °C detected by active surveillance or parasitemia and symptoms leading to treatment-seeking behavior in the absence of other clear cause of fever on passive surveillance. Children were enrolled as HC if they were asymptomatic for acute illness, had no evidence or history of chronic illness, and if the result of their thick blood smears was negative for malaria. All the subjects gave blood samples that were blotted onto Whatman filter papers FTA Classic cards and dried.

### Genetic analysis

Genomic DNA was isolated from punctures of the FTA cards (6 mm in diameter) incubated in 0.4 mL of the phosphate buffered saline (1X PBS), pH 7.4, for 20 min at room temperature with gentle agitation. Following centrifugation, the supernatant was discarded and the paper was treated with 40 μL of a solution containing 10 mM NaOH, 200 mM NaCl, and 0.05% sodium dodecyl sulfate for 6 min at 95°C. After centrifugation for 3 min, the supernatant was collected. Aliquots were diluted 12.5 X in water and used for PCR. TaqMan assays were used for genotyping the SNPs. The assay is based on the presence of fluorescence due to degradation of allele-specific fluorochrome-conjugated probes after annealing to SNP-containing PCR amplicon [32]. It was performed on a Stratagene Mx 3005P thermocycler instrument (Cedar Creek, TX). Table 1 describes the SNPs as well as the sequence context from which allele-specific fluorogenic probes were derived. Primers and fluorogenic probes were purchased from Applied Biosystems (Etobicoke, ON). The probes for common and minor alleles carried at their 5’-end a VIC and a FAM fluorochromes, respectively. They all carried a non-fluorescent quencher (NFQ) at their 3-end. A typical PCR reaction mixture contained 2 μL of DNA sample, 1x FastStart TaqMan ProbeMaster Rox master mix (Roche, Laval, QC), primers at 0.9 μM each and fluorogenic probes at 0.2 μM each. The reaction was run for 45–50 cycles involving a 15-20-sec denaturation at 95°C, a 20-sec annealing at the appropriate temperature, and 20-s elongation at 72°C.

**Table 1:** *PCSK9* SNPs under study.

SNP ID	c.#N_1_>N_2_ (p.AA_1_#AA_2_)^a^	Context Sequences: 5’-3’^b^	Exon	Phenotype^c^	MAF^d^	HWE^e^
rs28362263	c.1327G>A (p.A443T)	cccaacctggtggcc**[g/a]**ccctgccccccagca	8	LOF	0.12	0.23
rs562556	c.1420A>G (p.I474V)	cggatggccacagcc**[a/g]**tcgcccgctgcgccc	9	LOF	0.20	0.96
rs505151	c.2009A>G (p.E670G)	gcagcaccagcgaag**[a/g]**ggccgtgacagccgt	12	GOF	0.26	0.71
rs28362286	c.2037C>A (p.C679X)	cgttgccatctgctg**[c/a]**cggagccggcacctg	12	LOF	0.02	0.90

^a^ Codon.# common nucleotide>variant nucleotide (protein.common amino acid#variant amino acid).

^b^ The polymorphic nucleotides are written in bold and bracketed.

^c^ LDLR-degrading activity: loss-of-function (LOF) or gain-of-function (GOF).

^d^ Minor allele frequency in the whole population sample.

^e^ Hardy-Weinberg equilibrium.

### Statistical analysis

A preliminary quality control of all data was conducted (Supporting information: S1 Text). Conformity of each SNP to Hardy-Weinberg equilibrium (HWE) was independently evaluated using an online software (www.oege.org). Chi-square tests or Fisher’ exact tests were used to compare genotype distributions or allele frequencies between cases and controls. The strength of allelic association with malaria was expressed as odds ratios (OR) and 95% confidence intervals (95% CI). A *P* value of < 0.05 was set for significance in all analyses. The data were analyzed by GraphPad Prism 5 software.

## Results

A total of 752 DNA samples were genotyped for the rs28362263 (G>A), rs562556 (A>G), rs505151 (A>G), and rs28362286 (C>A) SNPs leading to A443T, I474V, E670G, and C679X PCSK9 polymorphisms, respectively. Their genotype distribution in this population sample did not deviate from HWE and their MAFs were 0.12, 0.20, 0.26, and 0.022, respectively (see Table 1).

Since we presumed that plasma cholesterol level could influence the vulnerability to malaria, we examined the frequency of these cholesterolemia-modifying *PCSK9* SNPs among healthy and malaria-stricken children in our cohort. The results per genotype and per health condition severity are presented in Table 2.

**Table 2:** Genotype distribution among healthy and malaria children.

SNP		Healthy Controls (HC, N = 253)			Uncomplicated Malaria (UM, N = 246)			Severe Malaria (SM, N = 253)			Statistics^b^	
	G:^a^	0	1	2	0	1	2	0	1	2	*P*_exact_	*P*_trend_
**A443T**	N	191	53	9	202	42	2	197	47	8	0.112	0.888
	%	75.5	20.9	3.6	82.1	17.1	0.8	78.2	18.7	3.2		
**I474V**	N	162	77	14	151	85	10	168	76	8	0.437	0.226
	%	64.0	30.4	5.5	61.4	34.6	4.1	66.7	30.2	3.2		
**E670G**	N	145	99	9	140	92	14	126	106	20	0.110	**0.035**
	%	57.3	39.1	3.6	56.9	37.4	5.7	50.0	42.1	7.9		
**C679X**	N	237	15	0	237	8	1	244	8	0	0.269	0.126
	%	94.1	5.9	0.0	96.3	3.2	0.4	96.8	3.2	0.0		

^a^ G, genotypes by number of variant allele: **0** homozygotes for the common allele; **1**, heterozygotes; **2**, homozygotes for variant allele; N, number of subjects; % of subjects per genotype.

^b^, *P* exact or trend was determined by Chi^2^, Strong trends among health conditions is shaded.

The percent of heterozygotes for the dominant 679X variant were 5.9%, 3.2, and 3.2% among HC, UM, and SM groups, respectively. Although the difference was not significant, the trend would be expected if low cholesterol offered relative protection against malaria. A similar trend was observed for homozygotes for the recessive 474V variant (HC/UM/SM: 5.5/4.1/3.2%), but not for homozygotes for the recessive 443T variant (HC/UM/SM: 3.58/0.81/3.16%). Interestingly, an opposite trend was noted for the recessive GOF 670G variant (HC/UM/SM: 3.6/5.7/7.9%, Chi-square for trend *P* = 0.035), suggesting that hypercholesterolemia may render individuals relatively more susceptible to severe malaria.

When we grouped all malaria cases and compared the genotype distribution and allele frequencies for each SNP, no association was observed (Supporting information: S2 Table). However, when we compared healthy controls to severe malaria cases or uncomplicated malaria, we noted a significant association of the GOF 670G variant with susceptibility to severe malaria (OR: 1.38; 95% CI: 1.04–1.83, *P* = 0.031) (Table 3), and a significant association of the LOF 443T variant with protection from uncomplicated malaria (OR: 0.63; 95% CI: 0.43–0.94, *P* = 0.024) (Table 4).

**Table 3:** Association analysis of PCSK9 SNPs and severe malaria.

SNP	(PCSK9)		Healthy Controls (N = 253)			Severe Malaria Cases (N = 253)			Statistics^b^			
		G:^a^	0	1	2	0	1	2	*P*_g_	*P*_a_	OR	96% CI
rs28362263	(A443T)		191	53	9	197	47	8	0.775	0.516	0.88	(0.61–1.26)
rs562556	(I474V)		162	77	14	168	76	8	0.417	0.340	0.85	(0.62–1.17)
rs505151	(E670G)		145	99	9	126	106	20	0.057	**0.031**	**1.38**	**(1.04–1.83)**
rs28362286	(C679X)		237	15	0	244	8	0	0.200	0.205	0.53	0.22–1.25)

^a^ G, genotypes by number of variant allele: **0**, homozygotes for common allele; **1**, heterozygotes; **2**, homozygotes for variant allele.

^b^ *P*_g_, statistical differences of genotypes distribution (Chi^2^ test); *P*_a_, statistical differences of allelic frequencies (Fisher’s exact test); OR, odds ratio; CI, confidence interval. Significant differences between cases and controls are shaded.

**Table 4:** Association analysis of PCSK9 SNPs and uncomplicated malaria.

SNP	(PCSK9)		Healthy Controls (N = 253)			Uncomplicated Malaria Cases (N = 246)			Statistics^b^			
		G:^a^	0	1	2	0	1	2	*P*_g_	*P*_a_	OR	(%CI)
rs28362263	(A443T)		191	53	9	202	42	2	0.051	**0.024**	**0.63**	**(0.43–0.94)**
rs562556	(I474V)		162	77	14	151	85	10	0.591	0.877	1.04	(0.76–1.41)
rs505151	(E670G)		145	99	9	140	92	14	0.513	0.605	1.09	(0.81–1.46)
rs28362286	(C679X)		237	15	0	237	8	1	0.297	0.302	0.61	(0.26–1.40)

^a^ G, genotypes by number of variant allele: **0**, homozygotes for common allele; **1**, heterozygotes; **2**, homozygotes for variant allele.

^b^ *P*_g_, statistical differences of genotypes distribution (Chi^2^ test); *P*_a_, statistical differences of allelic frequencies (Fisher’s exact test); OR, odds ratio; CI, confidence interval. Significant differences between cases and controls are shaded.

Interestingly, when the allele distribution of the E670G polymorphism was considered by gender, the association of the G variant with severe malaria was significant in males (OR: 2.70; 95% CI: 1.74–4.19; *P* < 0.0001), not in females (OR: 1.37; 96% CI: 0.89–2.11; *P* = 0.186). Indeed, 15 out the 20 homozygous carriers of the 670G variation (75%) were males. No such gender dichotomy was observed for the A443T polymorphism in relation with uncomplicated malaria (Supporting information: S3 Table). No association was detected when UM cases were compared to SM cases for any of the SNPs (see Supporting information: S2 Table). The E670G genotypes did not influence the manifestation of malaria symptoms (Supporting information: S4 Table), nor the levels of blood glucose, hemoglobin, white blood cells or parasites (Supporting information: S1 Fig).

## Discussion

The E670G polymorphism is located in the cysteine/histidine-rich domain C-terminal domain (CHRD) of PCSK9. This domain is required for the LDLR-degrading activity of the protein [33]. The substitution of the charged Glu by a neutral Glycine at position 670 increases this activity presumably by altering CHRD conformation. The E670G polymorphism has been associated with hypercholesterolemia and increased risk of coronary artery disease [34]. In adults, for unknown reasons, the association with high plasma cholesterol appears to be male-gender specific [35]. In our children study, homozygous carriers of the minor allele, were mostly males and there were more of them among severe malaria patients than among healthy controls. These observations call for further studies that include lipid profiling to verify whether E670G-linked chronic hypercholesterolemia is indeed associated with a greater risk of severe malaria. In patients under septic shock, the GOF 670G variation was associated with greater blood levels of pro-inflammatory cytokines and greater mortality [36]. In mice, increased PCSK9 activity was linked to inflammation and septic shock lethality; its deficiency reversed these adverse outcomes [36, 37]. Therefore it is possible that the E670G polymorphism contributes to the inflammatory responses that commonly accompany and sometimes aggravate malaria [38, 39].

It is intriguing that the risk allele of the E670G polymorphism should be found at such a high frequency (MAF: 0.26) if it is associated with child morbidity and mortality due to endemic malaria. As in the case of balanced polymorphisms [40], it could provide some yet unknown biological benefits to carriers. On the other hand, using long-range haplotype test on the *PCSK9* locus, Ding and Kullo [41] have observed that the ancestral (major) allele of the E670G SNP may have been under positive selection in African-Americans, not in European-Americans, suggesting that it may have been advantageous for the survival or the reproduction of their African ancestors.

It was expected that minor allele of the rs28362286 (C>A) nonsense SNP would be less frequent among severe malaria patients than among healthy controls. It was not to a statistically significant extent. This SNP was initially identified among African-Americans at MAFs of 0.018 [8, 18]; it was observed in Zimbabwe and West Africa at MAFs of 0.04 and 0.033, respectively [19, 42]. The transversion introduces a premature termination codon leading to the production of a truncated protein that is not secreted and thus is incapable of promoting LDLR degradation [43, 44]. It is associated with a remarkable 28% reduction of mean plasma cholesterol in heterozygotes [8, 9, 18, 42]. The lower MAF of this SNP would require a larger population sample to establish any protective effect against severe malaria.

The rs28362263 (G>A) and rs562556 (A>G) LOF SNPs are found in many populations at varying frequencies [9, 11, 12, 45, 46]. Their minor alleles encode 443T and 474V LOF PCSK9 variants, respectively. These alleles are recessive since they are associated with significant reduction of plasma LDL-C level only in homozygotes [9, 45, 46]. The 443T variant has been shown to be more susceptible to proteolytic inactivation by the furin convertase [44], explaining its LOF phenotype. Why it appears to protect against uncomplicated malaria and not against the severe form remains to be investigated.

## Conclusions

Data presented in this preliminary report support the notion that PCSK9 activity could affect susceptibility to malaria. This finding should be corroborated by prospectively studying a larger population sample. It should be pointed out that sub-Saharan Africans commonly carry more than one non-synonymous LOF and GOF *PCSK9* SNPs in various combinations [12]. In the present cohort, 17.6% of subjects carried two of the four SNPs examined. In such cases, the resultant phenotype could be difficult to predict unless they are correlated with cholesterolemia. A substantial deviation from normal cholesterolemia in the absence of malaria may be a valid predictor of susceptibility or resistance to it. Furthermore, induction of normocholesterolemia with anti-PCSK9 drugs in hypercholesterolemic individuals could be useful for anti-malaria prophylaxis and therapy.

## Supporting information

S1 Text Statistical analysis. (DOCX)

S1 Table Cohort age, gender et ethnicity. (DOCX)

S2 Table SNP association analysis HC vs all malaria and UM vs SM. (DOCX)

S3 Table Association analysis of E670G and A443T SNPs with malaria per gender. (DOCX)

S4 Table Malaria symptoms per E670G genotypes. (DOCX)

S1 Fig Blood parameters per E670G genotypes. Blood parameters per genotype of the rs505151 (A>G) PCSK9 SNP (E670G) in uncomplicated and severe malaria. No significant difference was observed among genotype within either malaria conditions.
(TIF)
