# Supplementary material for: Malaria severity: Possible influence of the E670G PCSK9 polymorphism: A preliminary case-control study in Malian children
Source: PLoS One. 2018 Feb 15;13(2):e0192850. doi: 10.1371/journal.pone.0192850 (PMC5813955; doi:10.1371/journal.pone.0192850)
Supplement: S1 Text — (DOCX) [file pone.0192850.s001.docx]

### S1 Text: Statistical Analysis

Before data analysis, the biostatistician performed the quality control of the data. Categorical variables were described as absolute numbers or percentages, and continuous variables are presented as medians and interquartile ranges (IQRs). We used a Kolmogorov–Smirnov test to determine the distribution of continuous variables. Fisher’s exact test was used for comparisons of categorical variables. For data with a normal distribution, an unpaired t test (Student’s t test) was used for determining continuous variables. The Mann–Whitney U test was used to determine differences between non-normally distributed continuous variables. The repeated measures general linear model (repeated measures analysis of variance [ANOVA]) was used to analyze variables with repeated measures. Correlation coefficients were determined according to Spearman’s rank correlation test. The data were analyzed using the STATA (Version 14.0) software.
