# Supplementary material for: Malaria severity: Possible influence of the E670G PCSK9 polymorphism: A preliminary case-control study in Malian children
Source: PLoS One. 2018 Feb 15;13(2):e0192850. doi: 10.1371/journal.pone.0192850 (PMC5813955; doi:10.1371/journal.pone.0192850)
Supplement: S1 Table — (DOCX) [file pone.0192850.s002.docx]

### S1 Table. Age, gender, ethnicity distribution among groups

|  |  |  | **HC** | |  | **UM** | |  | **SM** | |
| --- | --- | --- | --- | --- | --- | --- | --- | --- | --- | --- |
| N |  |  | 253 | |  | 246 | |  | 253 | |
| Age ( months): | Mean ± SD |  | 39.3 ± 27.3 | |  | 40.6 ± 28.1 | |  | 38.7 ± 27.6 | |
|  | Range: |  | 1 - 164 | |  | <1 - 171 | |  | <1 - 159 | |
| ≤ 66 months: | n, (%) |  | 215 | (85.0) |  | 209 | (85.0) |  | 215.0 | (85.0 |
| Gender, female: | n, (%) |  | 119 | (47.0) |  | 124 | (50.4) |  | 115 | (45.5 |
| Ethnicity: | n, (%) |  |  |  |  |  |  |  |  |  |
| Bambara |  |  | 4 | (1.6) |  | 8 | (3.3) |  | 9 | (3.6) |
| Bozo |  |  | 2 | (0.8) |  | 2 | (0.8) |  | 0 | (0.0) |
| Dogon |  |  | 213 | (84.2) |  | 207 | (84.1) |  | 201 | (79.4) |
| Haoussa |  |  | 1 | (0.4) |  | 2 | (0.8) |  | 0 | (0.0) |
| Kasonkhe |  |  | 1 | (0.4) |  | 0 | (0.0) |  | 1 | (0.4) |
| Malinke |  |  | 3 | (1.2) |  | 3 | (1.2) |  | 2 | (0.8) |
| Minyaka |  |  | 4 | (1.6) |  | 0 | (0.0) |  | 2 | (0.8) |
| Mossi |  |  | 0 | (0.0) |  | 2 | (0.8) |  | 3 | (1.2) |
| Peuhl |  |  | 3 | (1.2) |  | 12 | (4.9) |  | 11 | (4.3) |
| Samogo |  |  | 0 | (0.0) |  | 2 | (0.8) |  | 1 | (0.4) |
| Senoufo |  |  | 5 | (2.0) |  | 0 | (0.0) |  | 1 | (0.4) |
| Soninke |  |  | 8 | (3.2) |  | 0 | (0.0) |  | 3 | (1.2) |
| Sonrhai |  |  | 4 | (1.6) |  | 3 | (1.2) |  | 9 | (3.6) |
| Toucouleur |  |  | 1 | (0.4) |  | 2 | (0.8) |  | 3 | (1.2) |

HC, healthy controls ; UM, uncomplicated malaria patients ; SM, severe malaria patients.
