# Supplementary material for: Malaria severity: Possible influence of the E670G PCSK9 polymorphism: A preliminary case-control study in Malian children
Source: PLoS One. 2018 Feb 15;13(2):e0192850. doi: 10.1371/journal.pone.0192850 (PMC5813955; doi:10.1371/journal.pone.0192850)
Supplement: S2 Table — (DOCX) [file pone.0192850.s003.docx]

### S2 Table. Analysis of association of PCSK9 SNPs

A. With malaria

| **SNP** |  |  | **Healthy** **Controls** | | |  | **Malaria** **Cases** | | |  | **Statistics**^b^ | | | | | |
| --- | --- | --- | --- | --- | --- | --- | --- | --- | --- | --- | --- | --- | --- | --- | --- | --- |
|  |  |  | **(N = 253)** | | |  | **(N = 498)** | | |  |  |  |  |  |  |  |
|  |  | **G:^a^** | **0** | **1** | **2** |  | **0** | **1** | **2** |  | ***P*_g_** |  | ***P*_a_** |  | **OR** | **(95% CI)** |
| rs28362263 | (A443T) |  | 191 | 53 | 9 |  | 399 | 89 | 10 |  | 0.236 |  | 0.093 |  | 0.75 | (0.55-1.04) |
| rs562556 | (I474V) |  | 162 | 77 | 14 |  | 319 | 161 | 18 |  | 0.440 |  | 0.734 |  | 0.95 | (0.73-1.24) |
| rs505151 | (E670G) |  | 145 | 99 | 9 |  | 266 | 198 | 34 |  | 0.164 |  | 0.147 |  | 1.21 | (0.94-1.56) |
| rs28362286 | (C679X) |  | 237 | 15 | 0 |  | 481 | 16 | 1 |  | 0.083 |  | 0.191 |  | 0.60 | (0.30-1.20) |

B. With malaria severity

| **SNP** |  |  | **Uncomplicated Malaria** | | |  | **Severe Malaria** | | |  | **Statistics*** | | | | | |
| --- | --- | --- | --- | --- | --- | --- | --- | --- | --- | --- | --- | --- | --- | --- | --- | --- |
|  |  |  | **(N = 246)** | | |  | **(N = 253)** | | |  |  | | |  |  |  |
|  |  | **G: ^a^** | **0** | **1** | **2** |  | **0** | **1** | **2** |  | ***P*_at_** |  | ***P*_a_** |  | **OR** | **(%CI)** |
| rs28362263 | (A443T) |  | 202 | 42 | 2 |  | 197 | 47 | 8 |  | 0.144 |  | 0.128 |  | 1.39 | (.93-2.07) |
| rs562556 | (I474V) |  | 151 | 85 | 10 |  | 168 | 76 | 8 |  | 0.459 |  | 0.233 |  | 0.82 | (0.60-1.13) |
| rs505151 | (E670G) |  | 140 | 92 | 14 |  | 126 | 106 | 20 |  | 0.258 |  | 0.115 |  | 1.26 | (0.95-1.25) |
| rs28362286 | (C679X) |  | 237 | 8 | 1 |  | 244 | 8 | 0 |  | 0.804 |  | 0.205 |  | 0.52 | (0.22-1.25) |

^a^ G, genotypes by number of variant allele: **0**, homozygotes for common allele; **1**, heterozygotes; **2**, homozygotes for variant allele.

^b^ *P*_g_, statistical differences of genotypes distribution (Chi^2^ test); *P*_a_, statistical differences of allelic frequencies (Fisher’s exact test); OR, odds ratio; CI, confidence interval.
