# Supplementary material for: Malaria severity: Possible influence of the E670G PCSK9 polymorphism: A preliminary case-control study in Malian children
Source: PLoS One. 2018 Feb 15;13(2):e0192850. doi: 10.1371/journal.pone.0192850 (PMC5813955; doi:10.1371/journal.pone.0192850)
Supplement: S3 Table — (DOCX) [file pone.0192850.s004.docx]

**S2 Table. Association analysis of PCSK9 SNPs per gender**

A. With severe malaria

| **SNP** | **Gender** |  | **Healthy Controls** | | |  | **Severe Malaria Cases** | | |  | **Statistics^b^** | | |
| --- | --- | --- | --- | --- | --- | --- | --- | --- | --- | --- | --- | --- | --- |
|  |  |  | **(N = 253)** | | |  | **(N = 253)** | | |  |  | | |
| rs505151 (E670G) |  | **G:^a^** | **0** | **1** | **2** |  | **0** | **1** | **2** |  | ***P*_a_** | **OR** | **(95% CI)** |
|  | F: (n = 241) |  | 73 | 39 | 8 |  | 62 | 54 | 5 |  | 0.0754 | 1.52 | (0.98-2.35) |
|  | M: (n = 265) |  | 72 | 60 | 1 |  | 68 | 49 | 15 |  | < 0.0001 | 2.74 | (1.76-4.25) |

B. With uncomplicated malaria

| **SNP** | **Gender** |  | **Healthy Controls** | | |  | **Uncomplicated Malaria** | | |  | **Statistics^b^** | | |
| --- | --- | --- | --- | --- | --- | --- | --- | --- | --- | --- | --- | --- | --- |
|  |  |  | **( N = 253)** | | |  | **Cases (N = 246)** | | |  |  | | |
| rs28362263 (A443T) |  | **G:^a^** | **0** | **1** | **2** |  | **0** | **1** | **2** |  | ***P*_a_** | **OR** | **(95%CI)** |
|  | F: (n = 244) |  | 87 | 28 | 5 |  | 100 | 22 | 2 |  | 0.060 | 0.60 | (0.35-1.02) |
|  | M: (n = 255) |  | 104 | 25 | 4 |  | 103 | 19 | 0 |  | 0.059 | 0.59 | (0.32-1.07) |

^a^ G, genotypes by number of variant allele: **0**, homozygotes for common allele; **1**, heterozygotes; **2**, homozygotes for variant allele.

^b^ *P*_a_, statistical differences of allelic frequencies (Fisher’s exact test); OR, odds ratio; CI, confidence interval. F. female subjects; M, male subjects.
