# Supplementary material for: Malaria severity: Possible influence of the E670G PCSK9 polymorphism: A preliminary case-control study in Malian children
Source: PLoS One. 2018 Feb 15;13(2):e0192850. doi: 10.1371/journal.pone.0192850 (PMC5813955; doi:10.1371/journal.pone.0192850)
Supplement: S4 Table — (DOCX) [file pone.0192850.s005.docx]

### S4 Table. Symptoms per genotype ^a^: rs505151 (A>G); E670G

| **Symptoms** |  | **Severe Malaria** | | | | | | | |  | **Uncomplicated Malaria** | | | | | | | |
| --- | --- | --- | --- | --- | --- | --- | --- | --- | --- | --- | --- | --- | --- | --- | --- | --- | --- | --- |
| **Genotypes:** |  | **AA** | |  | **AG** | |  | **GG** | |  | **AA** | |  | **AG** | |  | **GG** | |
|  |  | **n** | **(%)** |  | **n** | **(%)** |  | **n** | **(%)** |  | **n** | **(%)** |  | **n** | **(%)** |  | **n** | **(%)** |
| Total |  | 126 | (100.0) |  | 106 | (100.0) |  | 20 | (100.0) |  | 140 | (100.0) |  | 92 | (100.0) |  | 14 | (100.0) |
| Fever |  | 122 | (96.8) |  | 104 | (98.1) |  | 20 | (100.0) |  | 136 | (97.1) |  | 88 | (95.7) |  | 14 | (100.0) |
| Vomiting |  | 64 | (50.8) |  | 54 | (50.9) |  | 12 | (60.0) |  | 63 | (45.0) |  | 37 | (40.2) |  | 7 | (50.0) |
| Convulsion |  | 61 | (48.4) |  | 46 | (43.4) |  | 10 | (50.0) |  | 2 | (1.4) |  | 1 | (1.1) |  | 1 | (7.1) |
| Abdominal pain |  | 19 | (15.1) |  | 15 | (14.2) |  | 5 | (25.0) |  | 31 | (22.1) |  | 16 | (17.4) |  | 3 | (21.4) |
| Diarrhea |  | 34 | (27.0) |  | 34 | (32.1) |  | 6 | (30.0) |  | 46 | (32.9) |  | 23 | (25.0) |  | 2 | (14.3) |
| Respiratory symptoms |  | 62 | (49.2) |  | 45 | (42.5) |  | 9 | (45.0) |  | 74 | (52.9) |  | 40 | (43.5) |  | 7 | (50.0) |
| Splenomegaly 1-5* |  | 43 | (35.5) |  | 23 | (22.5) |  | 9 | (52.9) |  | 35 | (29,4) |  | 33 | (41.8) |  | 6 | (54.5) |
| Headache |  | 43 | (34.1) |  | 48 | (45.3) |  | 7 | (35.0) |  | 53 | (37.9) |  | 37 | (40.2) |  | 7 | (50.0) |
| Lethargy |  | 33 | (26.2) |  | 24 | (22.6) |  | 4 | (20.0) |  | 1 | (0.7) |  | 1 | (1.1) |  | 0 | (0.0) |
| Coma |  | 27 | (21.8) |  | 24 | (23.5) |  | 2 | (10.0) |  |  |  |  |  |  |  |  |  |
| BCS < 5^†^ |  | 41 | (33.1) |  | 26 | (25.5) |  | 6 | (30.0) |  |  |  |  |  |  |  |  |  |
| Seizures |  | 48 | (38.7) |  | 34 | (33.3) |  | 8 | (40.0) |  |  |  |  |  |  |  |  |  |
| Obtundation |  | 12 | (9.7) |  | 12 | (11.8) |  | 3 | (15.0) |  |  |  |  |  |  |  |  |  |
| Prostration |  | 17 | (13.7 |  | 11 | (10.8) |  | 3 | (15.0) |  |  |  |  |  |  |  |  |  |
| Hyperparasitemia |  | 60 | (48.4) |  | 50 | (48.0) |  | 12 | (60.0) |  |  |  |  |  |  |  |  |  |
| Anemia |  | 19 | (15.3) |  | 9 | (8.8) |  | 5 | (25.0) |  |  |  |  |  |  |  |  |  |
| Respiratory distress |  | 8 | (6.3) |  | 5 | (4.9) |  | 1 | (5.0) |  |  |  |  |  |  |  |  |  |
| Hypoglycemia |  | 0 | (0.0) |  | 4 | (3.9) |  | 0 | (0.0) |  |  |  |  |  |  |  |  |  |
| Jaundice |  | 6 | (4.8) |  | 7 | (6.9) |  | 2 | (10.0 |  |  |  |  |  |  |  |  |  |

^a^ Some percentages are relative to number of subjects of whom clinical data were available.

* Severity of spleen enlargement, from absent (0), to mild (1) to massive (5), as estimated by abdominal palpation.

^†^ Blantyre coma scale based on motor and verbal responses as well as eye movement: 5 is normal; < 5 is not.
