# Supplementary figures and images for: Malaria severity: Possible influence of the E670G PCSK9 polymorphism: A preliminary case-control study in Malian children
Source: PLoS One. 2018 Feb 15;13(2):e0192850. doi: 10.1371/journal.pone.0192850 (PMC5813955; doi:10.1371/journal.pone.0192850)

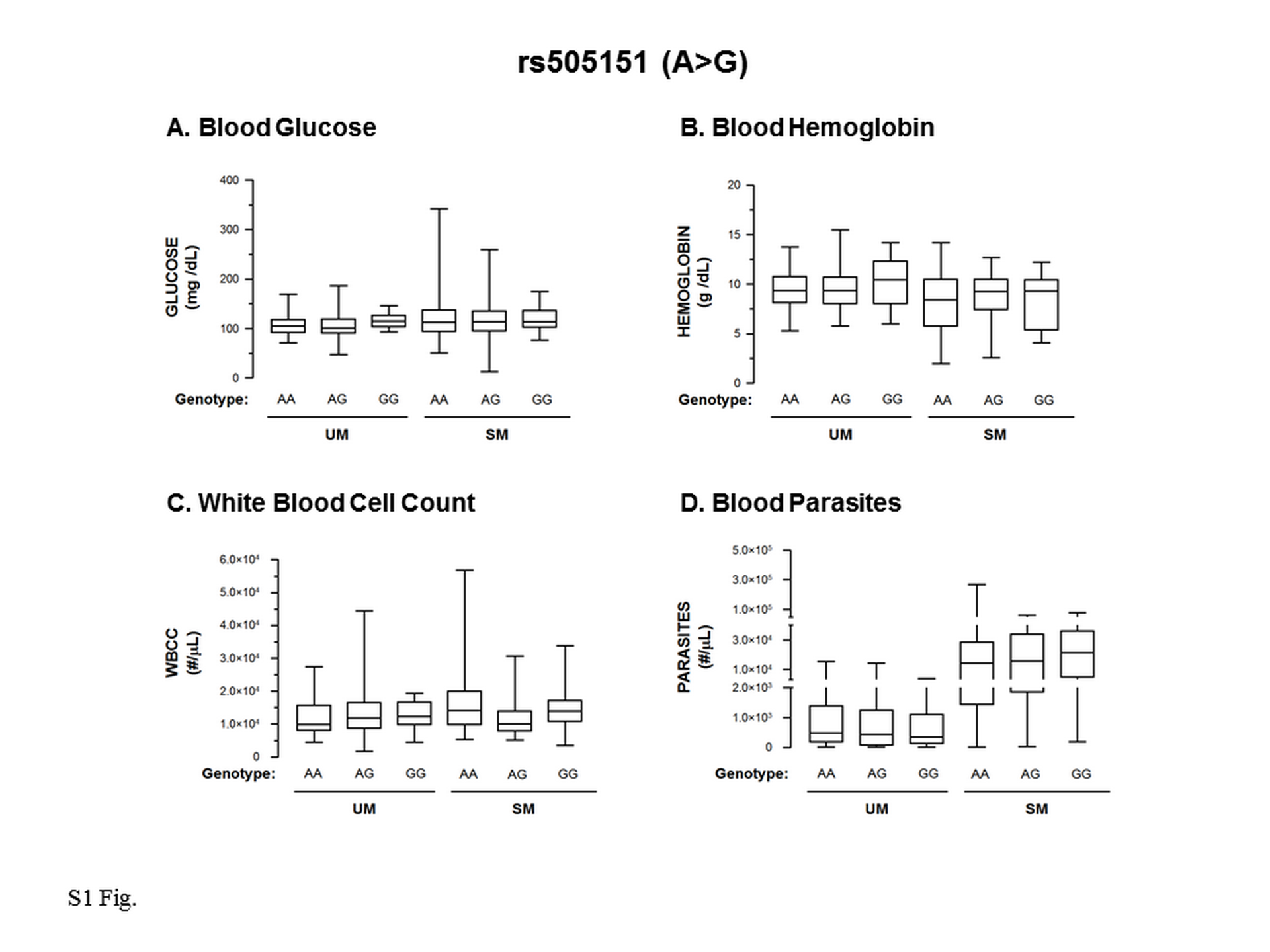

Supplement: S1 Fig — Blood parameters per genotype of the rs505151 (A>G) PCSK9 SNP (E670G) in uncomplicated and severe malaria. No significant difference was observed among genotype within either malaria conditions. (TIF) [file pone.0192850.s006.tif]
